# Estimation of Surface Heat Flux and Surface Temperature during Inverse Heat Conduction under Varying Spray Parameters and Sample Initial Temperature

**DOI:** 10.1155/2014/721620

**Published:** 2014-04-01

**Authors:** Muhammad Aamir, Qiang Liao, Xun Zhu, Aqeel-ur-Rehman, Hong Wang, Muhammad Zubair

**Affiliations:** ^1^Key Laboratory of Low-grade Energy Utilization Technologies and Systems, Chongqing University, Chongqing 400030, China; ^2^College of Computer Science and Engineering, Chongqing University, Chongqing 400030, China; ^3^Department of Basic Sciences, University of Engineering and Technology (UET), Taxila 47080, Pakistan

## Abstract

An experimental study was carried out to investigate the effects of inlet pressure, sample thickness, initial sample temperature, and temperature sensor location on the surface heat flux, surface temperature, and surface ultrafast cooling rate using stainless steel samples of diameter 27 mm and thickness (mm) 8.5, 13, 17.5, and 22, respectively. Inlet pressure was varied from 0.2 MPa to 1.8 MPa, while sample initial temperature varied from 600°C to 900°C. Beck's sequential function specification method was utilized to estimate surface heat flux and surface temperature. Inlet pressure has a positive effect on surface heat flux (SHF) within a critical value of pressure. Thickness of the sample affects the maximum achieved SHF negatively. Surface heat flux as high as 0.4024 MW/m^2^ was estimated for a thickness of 8.5 mm. Insulation effects of vapor film become apparent in the sample initial temperature range of 900°C causing reduction in surface heat flux and cooling rate of the sample. A sensor location near to quenched surface is found to be a better choice to visualize the effects of spray parameters on surface heat flux and surface temperature. Cooling rate showed a profound increase for an inlet pressure of 0.8 MPa.

## 1. Introduction

Water spray quenching of metals got a lot of attention due to its very high cooling efficiency. The traditional laminar quenching has low cooling rates in the range of 30°C/s~80°C/s [1], while spray quenching was reported to achieve high cooling rates in the range of 300°C/s~500°C/s depending on the thickness of the plate [2]. Spray cooling has been reported to achieve as high as 10 MW/m^2^ heat flux [3–5]. Spray characteristics like inlet pressure, mean velocity of the sprayed fluid, droplet size of the impinging fluid, mass flow rate of the fluid, nozzle to surface distance, type of nozzle, angle of spray, and degree of subcooling of the fluid greatly affect the cooling performance of the spray [6–9]. As there were many parameters involved in the characterization of a spray cooling performance, so there were enormous experimental and computational studies which had been conducted to get the overall theoretical understanding and potential application of spray cooling in different fields of technology [10, 11]. Due to complex nature of interaction of liquid and vapor phase, liquid impact, and phase change in spray cooling, it is difficult to understand the heat removal phenomena, so overall understanding of spray cooling is still in its infancy stages [3, 4]. Extensive experimental and computational work is still required to get the complete picture of spray cooling performance under different working conditions during spray quenching inverse heat conduction from metals.

Metal quenching is classified as ill-posed inverse heat conduction problem (IHCP). Direct chill (DC) casting is used to prepare the nonferrous metal ingots for the sheet metal rolling. In DC casting, heat transfer from the molten metal to the cooling water takes place through the outer solidified layer. Spray cooling derives large amount of heat by the evaporation of liquid droplets that are impinged upon a heated surface. In IHCP, boundary condition, that is, surface heat flux, of the quenched surface is to be estimated from the temperature measurements made at an arbitrary location inside the metal. Inside metal temperature histories are used due to practical difficulty to install and insulate the temperature sensor in the boundary of metal, associated with IHTP [12–14]. The estimation of heat flux (HF) demands the solution of the IHCP. Many researchers proposed different methods to estimate boundary heat flux for an IHTP, for example, Tikhonov regularization, iterative regularization, single future time step method, and the function specification method (FSM). A considerable number of contributions have been published considering combinations, modifications, and comparisons of these methods [15]. In present study Beck's function specification method was utilized to investigate the effect of spray parameter on the time-varying boundary surface condition of stainless steel sample during quenching process.

The mechanical properties of steel are directly related to microstructure of the steel which in return directly depend on the finish roll temperature and rate of cooling. In a typical production line of run out table of steel industry, the strips are reheated to a hot rolling temperature close to 900°C and then cooled down to coiling temperature of 600°C [16]. Cooling in this temperature range should develop multiphase microstructures to produce advance high quality steels. It is not possible to produce multiphase structures with conventional laminar cooling because creation of such structures requires very high cooling rate. Spray cooling technology has been reported to achieve such high cooling rates (~140°C/s for 6 mm and 300°C/s for a 4 mm thick carbon steel strip). Spray cooling with such high cooling rates is called ultrafast cooling [17, 18]. Ultrafast cooling (UFC) is supposed to be achieved if the multiplication product of plate thickness (mm) and cooling rate (°C/s) is greater than a threshold value of 800 [19, 20].

Current study focused on effects of inlet spray pressure, thickness of the sample, initial sample temperature, and sensor location on surface heat flux, surface temperature, and cooling rate during ultrafast inverse heat conduction from stainless steel plate utilizing Beck's sequential function specification method with one interior point temperature history.

## 2. Spray Parameters and Mathematical Model of IHCP

The effect of variation of inlet pressure on the mean volume diameter (MVD) can be determined by the following equation [21]:

(1) $$\begin{array}{c} d_{30}=\frac{9.5d_{n}}{\left(\Delta P^{0.37}\sin\theta /2\right)}, \end{array}$$

where *d*_30_, Δ*P*, *d*_*n*_, and *θ*  ( = 46°C) represent mean volume diameter, pressure drop between the nozzle and the spray chamber, the nozzle diameter, and the nozzle spray angle, respectively. The mean velocity, *u*_*o*_, of the spray droplets impinging on the test surface was calculated by using the following equation [16]:

(2) $$\begin{array}{c} u_{o}={\left(u_{j}^{2}+\frac{2\Delta P}{\rho_{l}}-\frac{12v}{\rho_{l}d_{30}}-2gz\right)}^{0.5}, \end{array}$$

where *u*_*j*_,  *v*, *ρ*_*l*_, *g*, and *z* represent spray velocity at nozzle exit, surface tension of the fluid, density of fluid, gravitational acceleration, and nozzle to surface distance, respectively. *u*_*j*_ can be calculated by simplifying Bernoulli equation [22] as follows:

(3) $$\begin{array}{c} u_{j}={\left(\frac{2P_{n}}{\rho_{l}}\right)}^{0.5}, \end{array}$$

where *P*_*n*_ represents nozzle pressure. The Sauter mean diameter (SMD), *d*_32_, was estimated by using the estimate *d*_32_ = 0.8 MVD [23]. The SMD, *d*_32_, was used to calculate the spray Weber number, *W*_*e*_, which was defined as

(4) $$\begin{array}{c} W_{e}=\frac{\rho_{l}u_{o}^{2}d_{32}}{v}. \end{array}$$

### 2.1. Direct and Indirect Problems

A direct problem is a common problem with known governing equation along with material properties involved in the equation. Initial and boundary conditions are per specified. The aim of the direct problem is to determine the field variable. On the other hand, in an inverse problem, a part of the usual description is not given and to be found. In inverse problem, some experimental data are given to estimate the unknown part of the usual description of the problem. Inverse problem can be categorized as a boundary inverse problem, coefficient inverse problem, and retrospective inverse problem. Present study deals with a boundary inverse problem. In a boundary inverse problem, boundary heat flux at *x* = 0 and/or the boundary heat transfer coefficient *h* at *x* = *L* are unknown and objective is to determine heat flux boundary condition as function of time, while material properties are supposed to be known with some experimentally measured temperature response at one or more points within body. Present inverse problem is to estimate the time-varying unknown boundary heat flux and surface temperature.

In order to estimate the surface heat flux history during the quenching process of metal sample, it is necessary to make a mathematical model of the heat conduction process. In present study, the quenched target was taken as homogeneous, isotropic, and flat. A mathematical illustration of the inverse heat conduction problem is given as [15, 24–26]

(5a) $$\begin{array}{c} \frac{\partial }{\partial x}\left(k\frac{\partial T}{\partial x}\right)=C\frac{\partial T}{\partial t}\;\text{for}\;\;x\in \left(0,L\right),\;\;t\in \left(0,t_{f}\right), \end{array}$$

(5b) $$\begin{array}{c} -k\frac{\partial T}{\partial x}=q\left(t\right)\;\text{for}\;\;x=0,\;\;t\in \left(0,t_{f}\right), \end{array}$$

(5c) $$\begin{array}{c} k\frac{\partial T}{\partial x}=h\left(t\right)\left[T\left(L,t\right)-T_{\infty }\right]\;\text{for}\;\;x=L,\;\;t\in \left(0,t_{f}\right), \end{array}$$

(5d) $$\begin{array}{c} T\left(x,0\right)=f\left(x\right)\;\text{for}\;\;x\in \left(0,L\right),\;\;t=0. \end{array}$$

The objective is to find

(6) $$\begin{array}{c} T\left(x,t\right)\;\text{for}\;\;t>0,\;\;0\leq x\leq L. \end{array}$$

Our target is indirect inverse heat conduction problem with unknown boundary heat flux at *x* = 0:

(7) $$\begin{array}{c} -k\frac{\partial T}{\partial x}=q\left(t\right). \end{array}$$

Boundary condition at *x* = *L* is

(8) $$\begin{array}{c} \frac{\partial q\left(t\right)}{\partial x}=0\;\text{or}\;h\left(t\right)=\text{constant}. \end{array}$$

Let us measure the temperature histories at an interior location of the sample which are given by

(9) $$\begin{array}{c} T\left(x_{j},t_{M}\right)=Y_{i,M}+\varepsilon_{j,M}\;,\;\;{\left\{x_{j}\right\}}_{j=1\ldots l}\subset \left(0,L\right),\\ {\left\{t_{M}\right\}}_{M=1\ldots M}\subset \left(0,t_{f}\right). \end{array}$$

Let us assume heat flux to be stepwise functions in the time interval (*t*_*M*−1_, *t*_*M*_). It is also assumed that the temperature history and heat flux are known at times *t*_*M*−1_, *t*_*M*−2_,… and the objective is to estimate *q*_*M*_ at time *t*_*M*_. Therefore (5b) can be written as

(10) q=−k∂T∂x={qM=Constant,for  (tM−1<t≤tM),q(t)=φ(t),for  (t>tM).

It is assumed that the unknown temperature field depends continuously on the unknown heat flux  *q*, let us denote *ϕ* = ∂*T*/∂*q* and differentiate (5a), (5b), (5c), and (5d) with respect to *q*; we get the direct problem

(11a) $$\begin{array}{c} \alpha \frac{\partial^{2}\phi }{\partial x^{2}}=\frac{\partial \phi }{\partial t}\;\;\;\text{for}\;\;x\in \left(0,L\right),\;\;t\in \left(0,t_{f}\right), \end{array}$$

(11b) $$\begin{array}{c} -k\frac{\partial \phi }{\partial x}=1\;\text{for}\;\;x=0,\;\;t\in \left(0,t_{f}\right), \end{array}$$

(11c) $$\begin{array}{c} k\frac{\partial T}{\partial x}=0\;\text{for}\;\;x=L,\;\;t\in \left(0,t_{f}\right), \end{array}$$

(11d) $$\begin{array}{c} \phi =0\;\text{for}\;\;x\in \left(0,l\right),\;\;t=0. \end{array}$$

At this point, let us introduce a new sensitivity coefficient, defined as

(12) $$\begin{array}{c} \phi_{i,m}^{M}={\frac{\partial T}{\partial q_{M}}|}_{\left(x_{i},t_{m}\right)}=\frac{\partial T_{i,m}}{\partial q_{M}}. \end{array}$$

The temperature *T*_*i*,*M*_ = *T*(*x*_*i*_, *t*_*m*_) can be expanded in a Taylor series about an arbitrary but known values of heat flux *q*_M_*. Considering the first-order derivatives, we get

(13) Ti,M=T^i,M+∂Ti,M∂qM|qM= q^M(qM−q^M)=T^i,M+ϕi,M(qM−q^M).

Solving (12) by using (10) and (11a), (11b), (11c), and (11d) for heat flux component *q*_*M*_ for temperature history only in one location *x*_1_, we get the formula

(14) $$\begin{array}{c} q_{M}={\hat{q}}_{M}+\frac{Y_{1,M}-{\hat{T}}_{1,M}}{\phi_{1,M}^{M}}. \end{array}$$

In case when future temperature measurements are used to estimate *q*_*M*_, we arrived to another formula [24–26]

(15) $$\begin{array}{c} q_{M}={\hat{q}}_{M}+\frac{\sum_{r=1}^{R}\left(Y_{1,M+r-1}-{\hat{T}}_{1,M+r-1}\right)\phi_{r}}{\sum_{r=1}^{R}{\left(\phi_{r}\right)}^{2}}. \end{array}$$

For only one point temperature (14) can be written as

(16) $$\begin{array}{c} q_{M}={\hat{q}}_{M}+\frac{\sum_{r=1}^{R}\left(Y_{M+r-1}-{\hat{T}}_{M+r-1}\right)\phi_{r}}{\sum_{r=1}^{R}{\left(\phi_{r}\right)}^{2}}, \end{array}$$

where ${\hat{T}}_{M+r-1}^{}=\sum_{i=1}^{M+r-1}{\hat{q}}_{i}\Delta \phi_{M+r-i-1}+T_{0}$.

For *r* = 1, (15) can be reduced to (13) which is called Stolz algorithm because Stolz [15] was the first to apply Duhamel's theorem to the IHCP for the single sensor single future temperature case. A MATLAB code for Beck's sequential function specification algorithm for one-dimensional IHCP with one interior point temperature history was used to estimate the surface heat flux and surface temperature.

## 3. Experimental Arrangements

**Figure 1:**
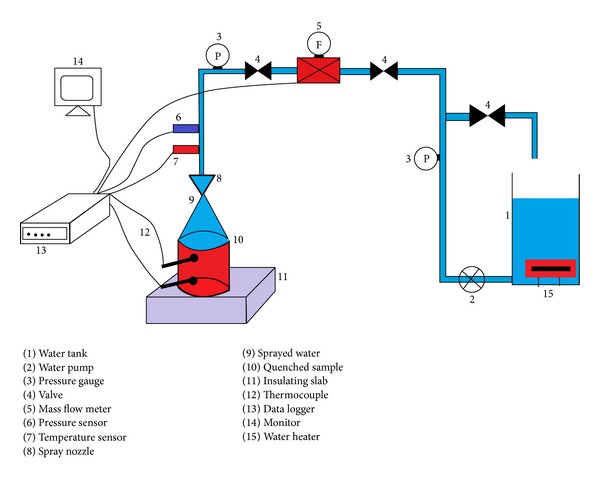
Sketch of experimental facility.

The experimental setup used in this research was comprised of three main systems, namely, fluid supply system, instrument system, and heating system, as shown in Figure 1.

### 3.1. Fluid Supply System

Fluid delivery system (FSS) consisted of a spray nozzle supplied by Spray Systems Co. Ltd. FSS was equipped with a CDL3-36 non-self-priming vertical multistage centrifugal pump with a head of 152 m. It can work in the fluid working temperature limits of −15°C to +120°C.

Coriolis mass flow meter (ZLJ series) had been used in the fluid delivery system to measure the mass flow rate of the fluid during spraying process. FSS had also been provided with a pressure sensor (0~2.5 MPa) and temperature sensors (K type thermocouples) to measure the pressure and temperature of the fluid in the FSS. A bypass valve had been provided in the FSS to control the inlet pressure of the spray nozzle. The FSS was connected with a water tank to supply the water. Water tank (capacity: 50 gallon) was equipped with 4 heaters to vary the inlet fluid temperature.

### 3.2. Instrumentation System

Instrumentation system includes all of the necessary electronic equipment to drive the FSS, to power heaters, and to acquire necessary measurements. It consists of a data acquisition system installed in personal computer, thermocouples installed at different geometrical locations inside the stainless steel sample, and FSS delivery system to monitor the temperature variations.

### 3.3. Heating System Fabricated Hot Surface

**Figure 2:**
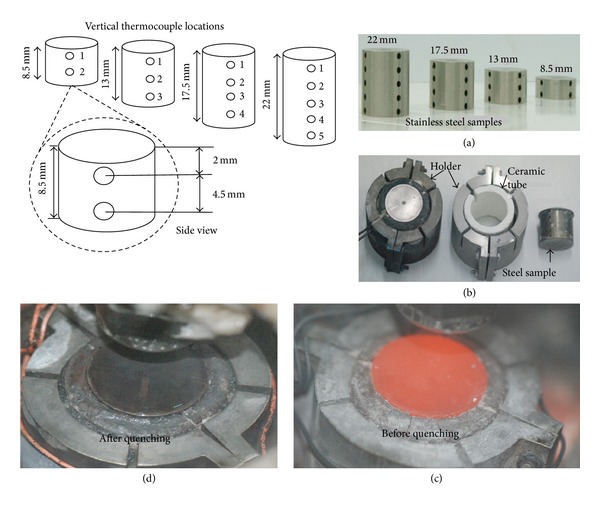
Details of cylindrical stainless steel samples: (a) stainless steel sample, (b) holder assembly, (c) red hot sample before quenching, and (d) sample after quenching.

The primary component of hot surface is a stainless steel sample (cylindrical shape) with a diameter *D* = 27 mm and thickness *δ*: 8.5 mm, 13 mm, 17.5 mm, and 22 mm. Four stainless steel plates of above-mentioned thicknesses were used in present study. Thermocouples were installed along the diameter of cylindrical block. The diameter of each thermocouple hole was *φ* = 2 mm. The vertical distance between 2 adjacent thermocouple holes on the same vertical line was 4.5 mm and the distance from the middle of hole to plate surface was 2 mm as shown in the side view of the heater in Figure 2.

In present work, we studied 1D spray heat transfer from top surface of the heater so cylindrical surface of the heater was insulated with ceramic tube and bottom surface was insulated with fiber glass insulation. Benson burner with natural gas was used to heat up the block to desire high temperature (100~900°C).

## 4. Results and Discussion

### 4.1. Effect of Inlet Pressure on Spray Parameters

**Figure 3:**
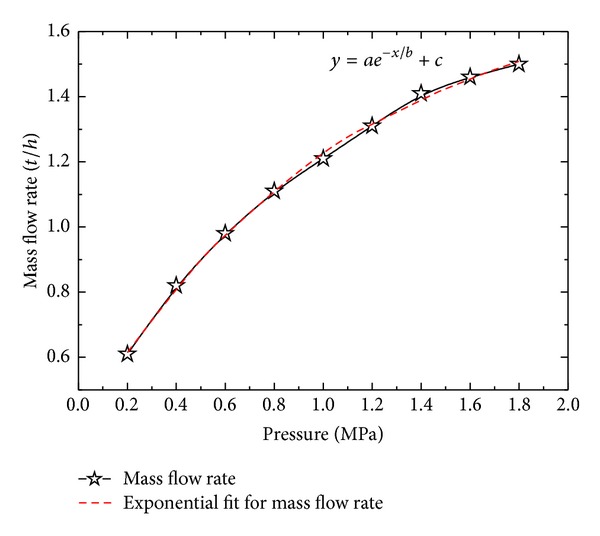
Variation of mass flow rate with inlet pressure.

**Figure 4:**
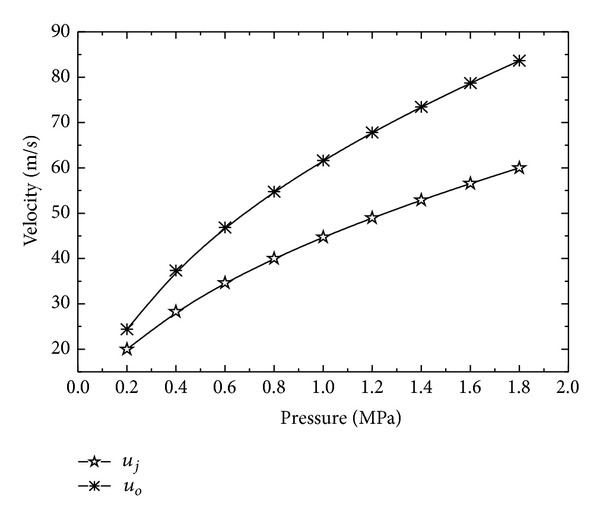
Variation of droplet velocity with inlet pressure.

**Figure 5:**
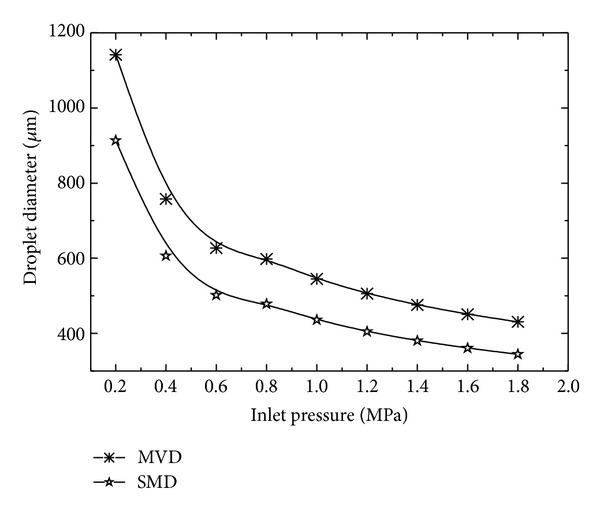
Variation in droplet size with inlet pressure.

**Figure 6:**
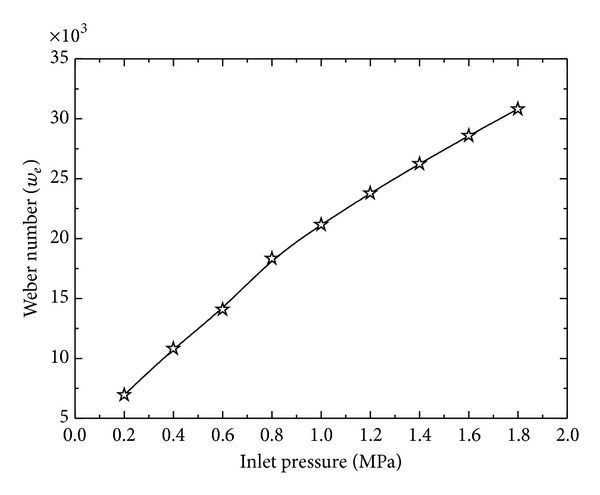
Variation of Weber number as a function of inlet pressure.

It is obvious that the flow rate of the water through nozzle increases with the increase in the inlet pressure. The increase in the mass flow rate is exponential as shown in Figure 3, which suggests the existence of a saturation value of flow rate beyond which it cannot increase even with a corresponding increase in the inlet pressure. An exponential fit equation of the following form is obtained:

(17) $$\begin{array}{c} y=ae^{-x/b}+c, \end{array}$$

where coefficient *a* = −1.75687 and constants *b* = 1.05023 and *c* = 1.75687 for present case.  Figure 4 shows that mean velocity and nozzle exit velocity also increase with the increase in the inlet pressure. At small inlet pressure, *u*_*o*_ and *u*_*j*_ values are close to each other, but *u*_*o*_ dominates as the inlet pressure increases. *u*_*o*_  and *u*_*j*_ increase exponentially suggesting an upper limit for the mean velocity and nozzle exit velocity for increasing inlet pressure. Mean volume diameter (MVD) and Sauter mean diameter (SMD) show an exponential decrease with increasing inlet pressure. Shear force causes the liquid to break apart into droplets and size of droplets decreases with increasing shear force for increasing inlet pressure [27]. The effect of inlet pressure on MVD and SMD is shown in Figure 5. For a wide range of inlet pressure, Weber number (*w*_*e*_) is a linear function of the inlet pressure (Figure 6).

### 4.2. Effect of Inlet Pressure on Surface Heat Flux and Surface Temperature

**Figure 7:**
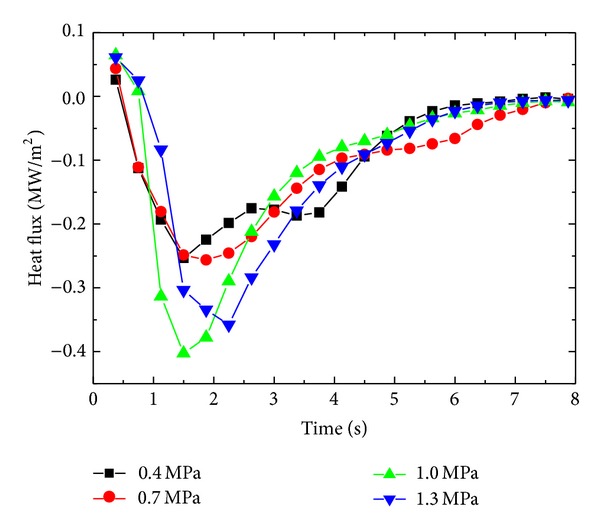
Variation of surface heat flux with inlet pressure.

**Figure 8:**
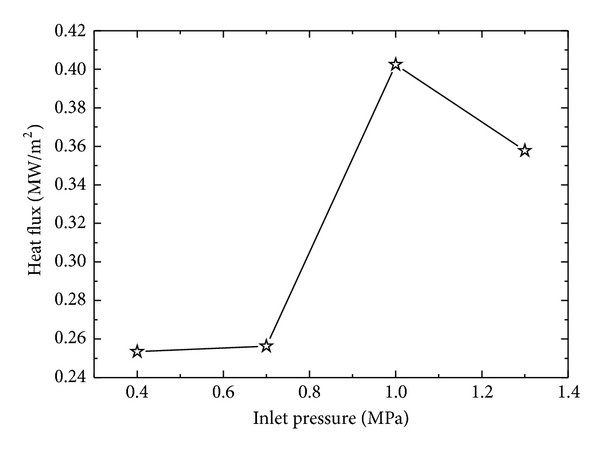
Variation of maximum surface heat flux as a function of inlet pressure.

In order to study the effect of inlet nozzle pressure on surface heat flux during spray cooling of stainless steel block of *δ* = 8.5 mm, inlet pressure is varied from 0.4 MPa to 1.3 MPa in steps of 0.3 MPa. An increasing trend in the surface heat flux is observed with the increase in inlet pressure up to 1.0 MPa. The maximum surface heat flux (MSHF) achieved for 0.4 MPa, 0.7 MPa, and 1.0 MPa is 0.2534 MW/m^2^, 0.2563 MW/m^2^, and 0.4024 MW/m^2,^ respectively. At inlet pressure of 1.3 MPa, the MSHF decreases to 0.3577 MW/m^2^. The decrease in the MSHF at high inlet pressure of the fluid can be explained by considering the effect of inlet pressure on mean velocity, *u*_*o*_, and droplet size of the water. The mean velocity, *u*_*o*_, of the spray droplets impinging on the test surface increases, while droplet size decreases with increasing inlet pressure as discussed in Section 4.1. These two important parameters have significant effect on the surface heat flux of the samples. Initial increase in the inlet pressure shortens the time required to cool the test surface to fluid temperature. After a threshold inlet pressure of 1.0 MPa, surface heat flux has a decreasing trend. It can be explained by considering the effect of mean droplet velocity and droplet diameter on the cooling performance of the fluid. Small size droplets are more efficient in removing the heat from a hot surface, while it is needed for such droplet to have sufficient residing time on the hot surface to absorb heat from the surface to fully evaporate. On the other hand, in present case, droplet size is decreased by increasing the inlet pressure of the fluid, which in response increases the mean droplet velocity. To a certain limit of inlet pressure, mean droplet velocity has positive effect on surface heat flux with decreasing mean droplet size. But after a critical value of pressure, the mean velocity has negative effect on surface heat flux due to the fact that the residing time of the droplet on the hot surface decreases with increasing velocity of the droplet. Another factor which affects the cooling efficiency of the droplet is the splashing and rebound of the droplet from the surface due to higher pressure and velocity of the droplet which have negative impact on the residing time of the droplet on the surface causing an associated decrease in MSHF from the test block. Figures 7 and 8 show the effect of inlet pressure on the surface heat flux of the quenched stainless steel block of *δ* = 8.5 mm.

**Figure 9:**
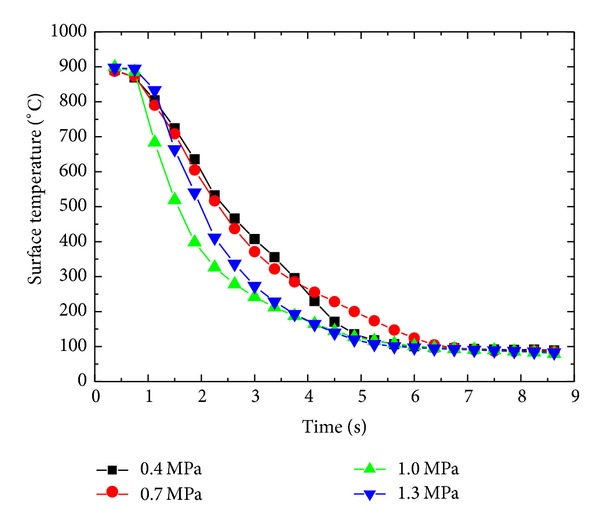
Variation of surface temperature with inlet pressure.

**Figure 10:**
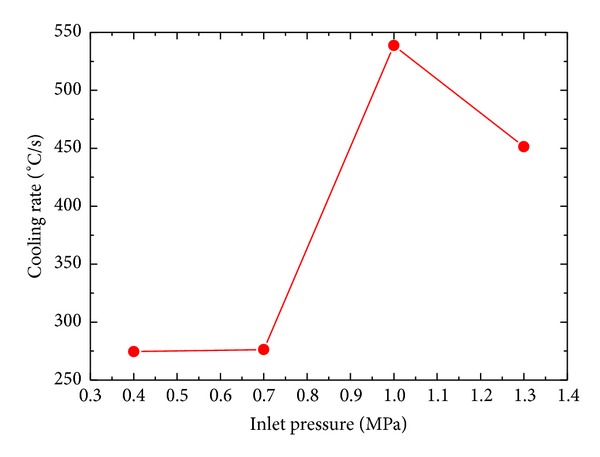
Effect of inlet pressure on cooling rate.

Figure 9 shows the variation of surface temperature with time for different inlet pressure. Cooling rate increases with increase in inlet pressure. Maximum cooling rates are achieved in the temperature range of 900°C~600°C. Cooling rates of 274.51°C/s, 276.31°C/s, 538.67°C/s, and 451.36°C/s are achieved for 0.4 MPa, 0.7 MPa, 1.0 MPa, and 1.3 MPa, respectively, as shown in Figure 10. Lower inlet pressure has little effect on the cooling rate of the surface. Cooling rate decreases after a critical value of inlet pressure. The cooling rates achieved during current spray cooling study fall well above the UFC criteria described in Section 1.

### 4.3. Effect of Thickness of Sample on Surface Heat Flux and Surface Temperature

The amount of heat content in any plate depends upon its dimension and initial temperature. In the present study, heat transfer is considered along the thickness of the plate, and in another direction it is supposed to be negligible. Hence, the plate thickness decides the heat content in the present case.

**Figure 11:**
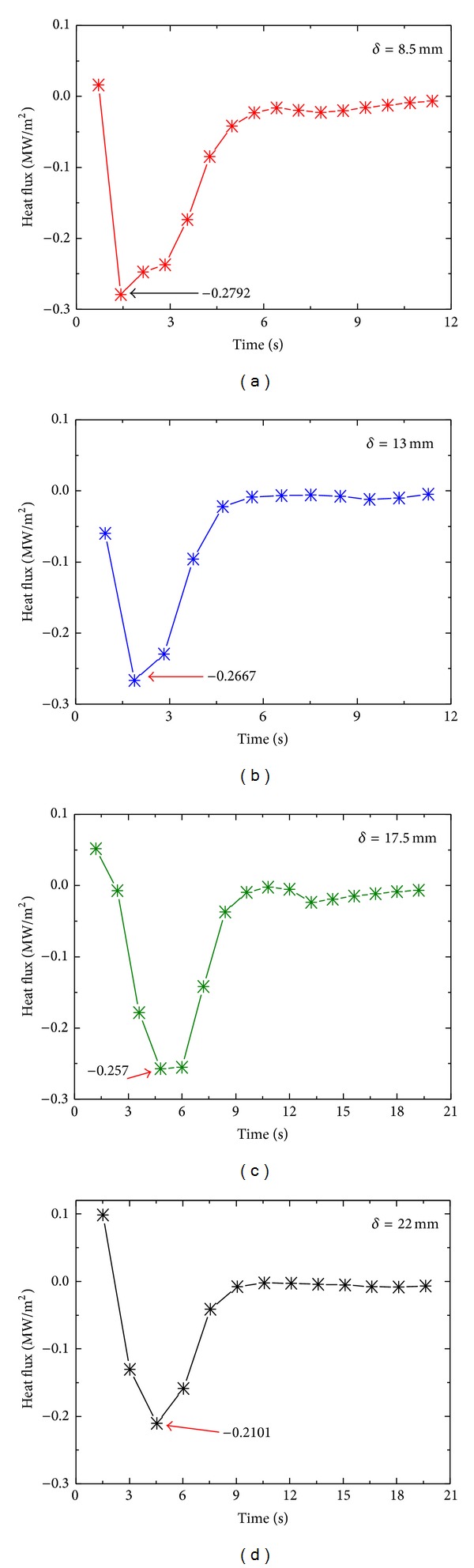
Variation of surface heat flux with thickness of the sample.

**Figure 12:**
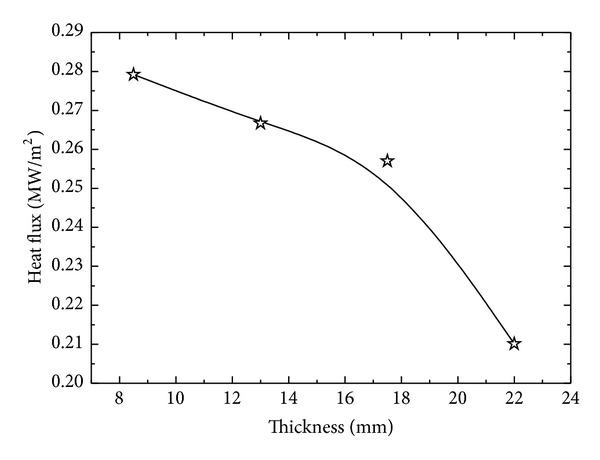
Variation of maximum surface heat flux with thickness of the sample.

It is shown in Figures 11(a), 11(b), 11(c), and 11(d) that maximum surface heat flux during spray quenching of the samples decreases with increasing thickness of the sample. The estimated values of maximum surface heat flux, for 8.5, 13, 17.5, and 22 mm thick plates, are 0.2792 MW/m^2^, 0.2667 MW/m^2^, 0.257 MW/m^2,^ and 0.2101 MW/m^2,^ respectively, showing decrease in surface flux (Figure 12) with increasing thickness of the sample.

**Figure 13:**
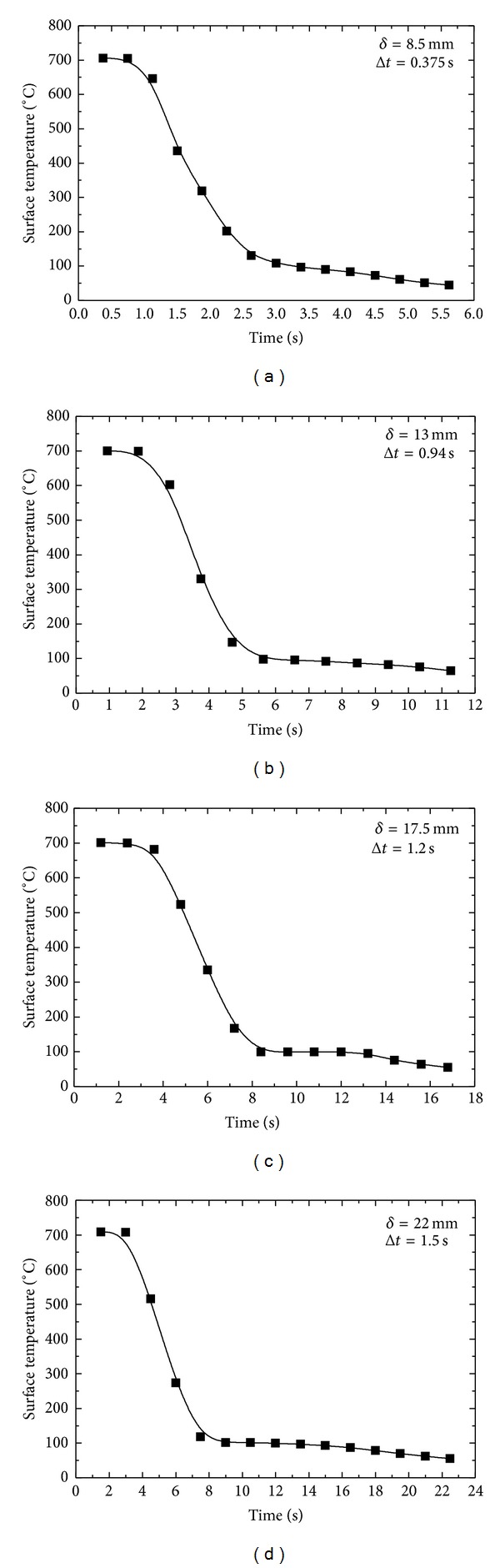
Variation of surface temperature with thickness of the sample.

The real-time surface temperature plots for *δ* = 8.5, 13, 17.5, and 22 mm thick samples are represented in Figures 13(a), 13(b), 13(c), and 13(d) at an inlet nozzle pressure of 1 MPa. As the thickness of the sample increases, the total amount of heat contained in the body also increases. In other words heat energy is distributed in a larger area. When such a sample at higher energy is subjected to water quenching, heat flows from the interior of the body to the surface subjected to force conviction due to water spray. As a sample with bigger dimensions contains bigger amount of heat, it in return helps keep the surface to be at higher temperature for longer time causing an associated decrease in cooling rate of the sample. But in the case of a thin plate, the surface temperature is high for a shorter duration of time, and, hence, the cooling rate is more than that of a thicker plate. The cooling rate has direct relation with surface flux. Higher cooling rates give higher surface heat flux.

**Figure 14:**
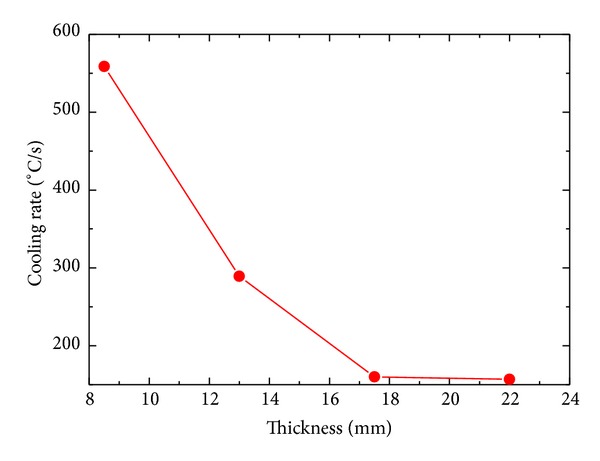
Effect of thickness sample on surface cooling rate.

As explained earlier, cooling rate decreases with increasing thickness of a sample. Cooling rates of 558.71°C/s, 289.11°C/s, 160.02°C/s, and 156.95°C/s are achieved for *δ* = 8.5 mm, 13 mm, 17.5 mm, and 22 mm, respectively, as shown in Figure 14. Cooling rate of sample decreases exponentially as a function of thickness of the plate.

### 4.4. Effect of Initial Sample Temperature on Surface Heat Flux and Surface Temperature

**Figure 15:**
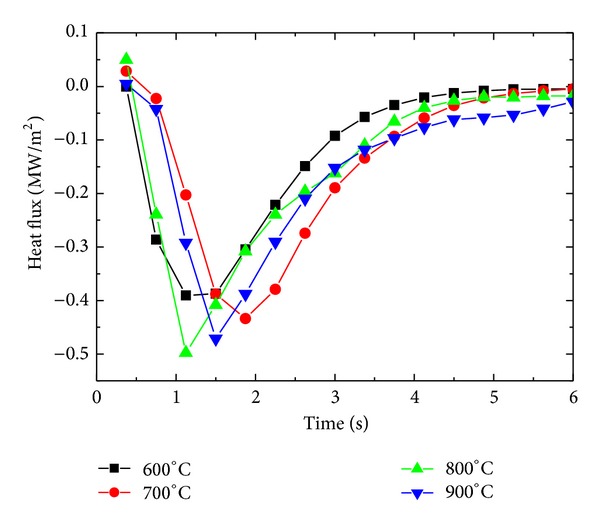
Variation of surface heat flux with initial average sample temperature.

**Figure 16:**
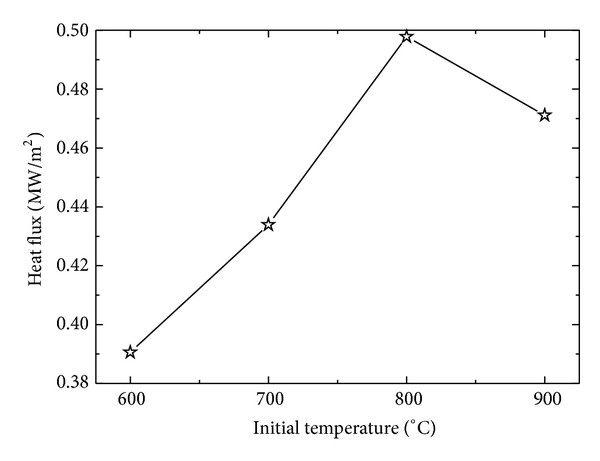
Variation of surface heat flux with initial sample temperature.

**Figure 17:**
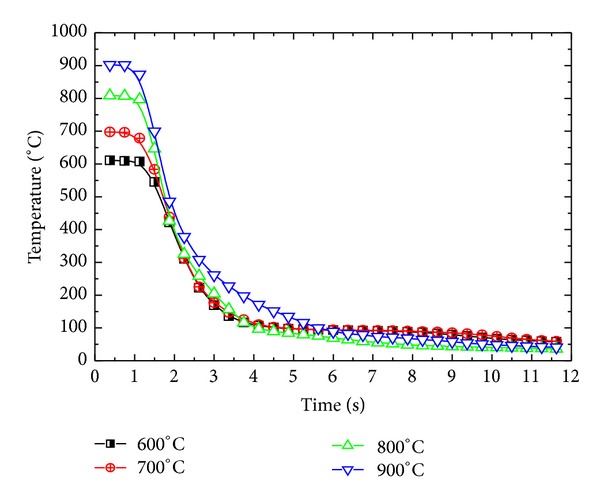
Variation of surface temperature with initial average sample temperature.

**Figure 18:**
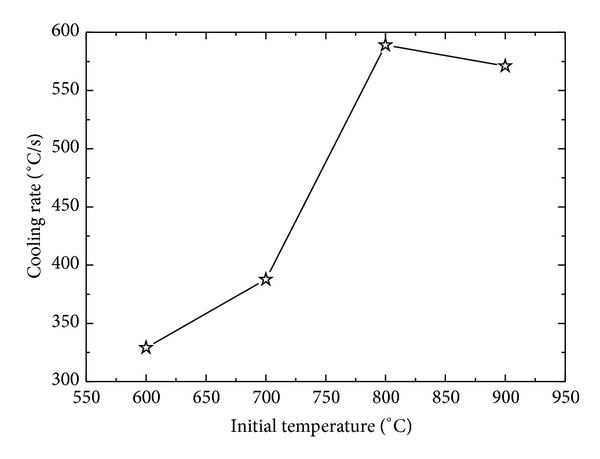
Variation of cooling rate with initial sample temperature.

Surface heat flux for four different initial sample temperatures was estimated under constant inlet pressure of 1 MPa for a sample of *δ* = 8.5 mm to observe the effects of film boiling on inverse heat conduction form the sample.  Figure 15 represents the variation of surface heat flux under different initial temperatures of the sample. For initial sample temperatures of 600°C, 700°C, and 800°C, maximum surface heat flux showed an increasing trend from 600°C to 800°C, that is, 390600 W/m^2^< 433900 W/m^2^< 497800 W/m^2,^ while, for initial sample temperature of 900°C, a corresponding decrease in maximum achieved heat flux was observed, that is, 471100 W/m^2^. The trend of maximum surface flux variation for different initial sample temperatures is represented in Figure 16. The decrease in the maximum flux can be attributed to the boiling film phenomena, which serves as a thermal insulation between the sample surface and the fluid. At initial sample temperature of 900°C, the film boiling phenomena become dominant and severely affect the cooling performance of the spray under present working conditions.  Figure 17 shows the cooling history of the surface for different initial sample temperatures. Film boiling screening can also be observed from the surface temperature history for initial temperature of 900°C. The maximum cooling rate shows a slight decrease for initial temperature in the range of 900°C as shown in Figure 18.

### 4.5. Estimation of Surface Heat Flux and Surface Temperature from Different Sensor Location

**Figure 19:**
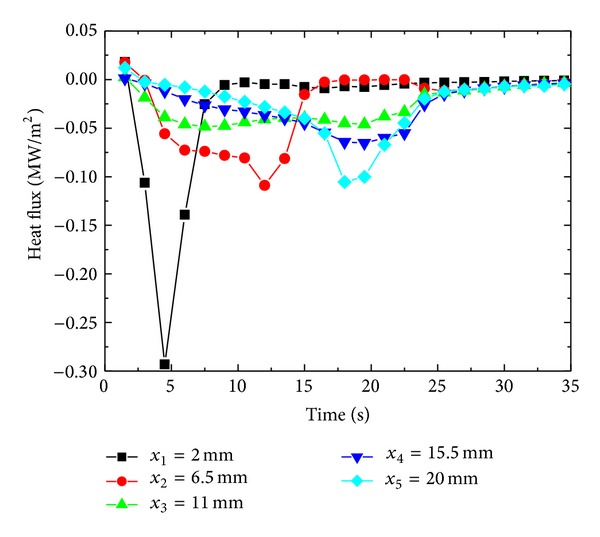
Estimation of surface heat flux from different sensor locations.

**Figure 20:**
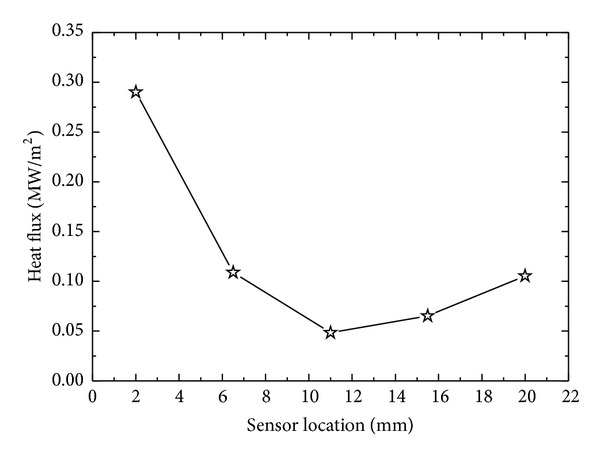
Variation of surface heat flux with different sensor locations.

**Figure 21:**
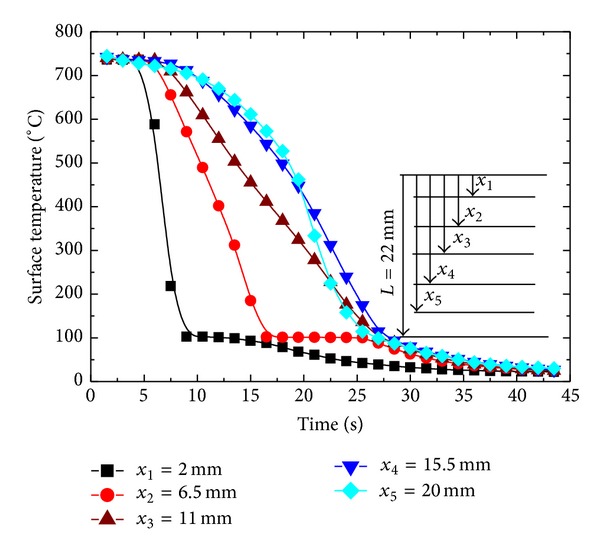
Estimation of surface temperature from different sensor locations.

**Figure 22:**
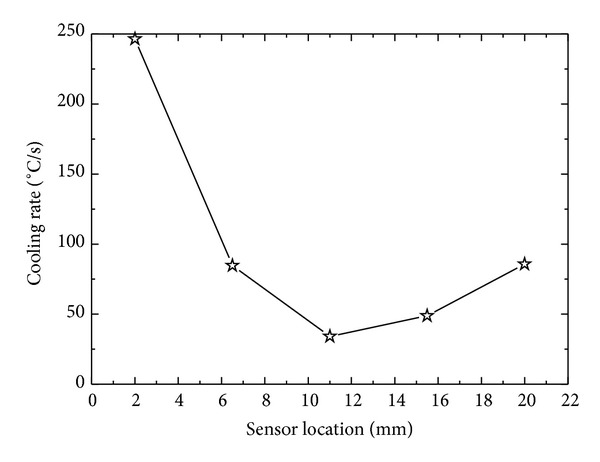
Variation of cooling rate with different sensor locations.

Though in present study we consider ID heat conduction but in real world no ideal thermal insulation exists which can totally prevent heat conduction from cylindrical and bottom surface of the cylinder. The temperature response at the surface from an interior point has an obvious dependence on the location of the temperature sensor. Estimated surface heat flux from the temperature response from different vertical locations from the sprayed cooled surface is shown in Figure 19. The sensor at *x*_1_ = 2 mm, being nearest to quenched surface, experiences maximum effect of quenching and gives a maximum temperature response to quenching. At *x*_1_, radial gradient of temperature is supposed to be minimum as temperature response is maximum in upward vertical direction at this location. Estimated surface heat flux from sensor locations *x*_2_ and *x*_3_ decreases with relative decrease in the temperature response at these locations. There is a relative increase surface flux estimated from *x*_4_ and *x*_5_ (Figure 20). It can be explained by considering the fact that the bottom surface of the surface is not ideally insulated; a part of heat might escape from the bottom surface of the cylindrical sample giving a pseudo enhanced temperature response to quenching from the top surface causing relatively high estimates of surface flux. It is found that sensor location nearest to quenched surface is a better option to observe the effects of spray and geometrical parameters on surface heat flux and surface temperature. Corresponding surface temperature histories estimated by utilizing temperature data from different sensor locations are shown in Figure 21. Surface cooling estimation follows the same trend as that of surface flux (Figure 22). It is due to that fact that surface temperatures were estimated by previously estimated surface flux utilizing Beck's sequential function specification method of inverse heat conduction causing a direct dependence of surface cooling rates on estimated surface flux at the end of each time step.

### 4.6. Effect of Pressure on Cooling Rate

**Figure 23:**
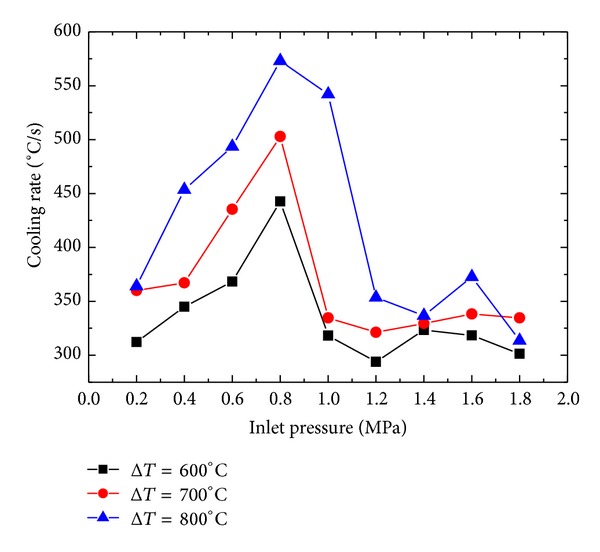
Effect of inlet pressure on surface cooling rate.

**Figure 24:**
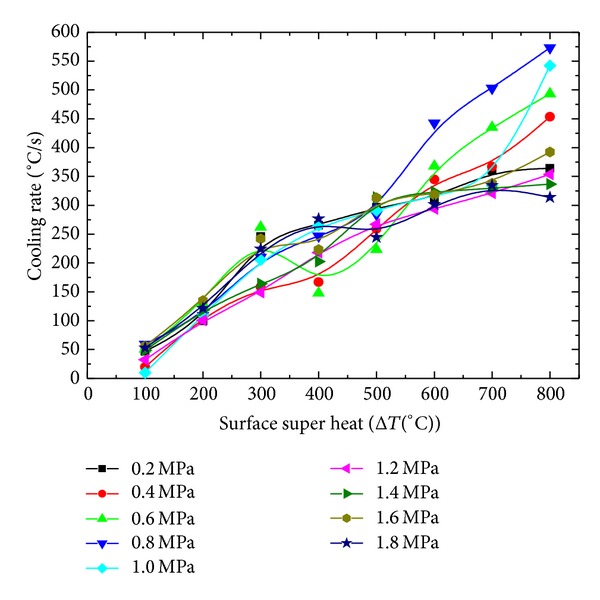
Effect of initial average body temperature on surface cooling rate.

As it is clear from Figures 4 and 5, inlet pressure affects the droplet size and velocity, so it is obvious that inlet pressure will also affect the cooling rate of the sample. The effect of inlet pressure on the cooling rate of the sample is shown in Figure 23. Cooling rate increases with the increase in the inlet pressure until 0.8 MPa. When inlet pressure is further increased to 1.0 MPA, a sudden decrease in the cooling rate is observed. Further increase in the inlet pressure shows little effect on the cooling rate. This behavior can be explained by considering the combined effect of the droplet size and droplet velocity on the cooling performance of the spray. In the lower pressure range, increasing droplet velocity along with depressing droplet size helps the droplet to penetrate through the insulting vapor film, developed at the heated surface due to evaporation of the fluid, causing effective contact between the droplet and the heated surface. An effective contact between the surface and the droplet results in a better cooling performance of spay, which results in an increased cool rate. At higher pressure range, there is a negative effect of velocity on the effective contact time of droplet to the sample surface. Effective contact time of the droplet decreases with increasing velocity of the droplet, which in return decreases the cooling efficiency of the spray. Another aspect, which might have a significant cause of lower cooling rates at higher pressure range, is relatively small droplet size. High temperature (600°C~900°C) of hot surface may cause the droplets of very small size to evaporate at vapor film surface before effective and actual contact of droplet with the hot surface. Earlier evaporation of droplets at the vapor film surface might be another cause of decrease in the cooling rate at higher inlet pressure range. It is obvious that cooling rate of the sample increases as the surface super heat increases as shown in Figure 24.

## 5. Conclusion

Present study was focused on determining the effects of inlet pressure, sample thickness, and sensor location on surface heat flux, surface temperature, and cooling rate during water quenching of stainless steel. Inverse heat conduction ill-posed Beck's sequential function specification linear method was used to estimate the time-varying surface heat flux and surface temperature of the quenched sample. A prior study was carried out to determine the effect of the inlet pressure on mass flow rate, mean spray velocity, inlet velocity, droplet size, and Weber number (*W*_*e*_). It was concluded that

1. cooling rates in the ultrafast cooling range were achieved with water spray cooling during quenching of the stainless steel samples;
2. maximum achieved surface heat flux increases with increase in inlet pressure, but this increase in surface heat flux is limited to a critical value of inlet pressure depending on a balance between the mean spray velocity and droplet size;
3. maximum achieved surface heat flux decreases with an increase in the thickness of the sample. In other words thickness of the plate has inverse relation with surface heat flux during quenching of the metal samples;
4. vapor film effects become dominant in the initial sample range of 900°C, causing a corresponding decrease in the maximum surface heat flux and cooling rate of the sample;
5. temperature sensor location near to quenched surface is a better choice to better understand the effects of spray parameters on the surface flux, surface temperature, and surface cooling rates;
6. the increase in inlet pressure increases cooling rate of the surface of the sample to a certain critical value of the inlet pressure depending on the individual characteristics of the nozzle to be used. Above the critical value of inlet pressure, cooling rate of the surface has decreasing trend;
7. surface cooling rate increases with increasing surface super heat.
